# Distinct virulence of Rift Valley fever phlebovirus strains from different genetic lineages in a mouse model

**DOI:** 10.1371/journal.pone.0189250

**Published:** 2017-12-21

**Authors:** Tetsuro Ikegami, Aaron Balogh, Shoko Nishiyama, Nandadeva Lokugamage, Tais B. Saito, John C. Morrill, Vinay Shivanna, Sabarish V. Indran, Lihong Zhang, Jennifer K. Smith, David Perez, Terry L. Juelich, Igor Morozov, William C. Wilson, Alexander N. Freiberg, Juergen A. Richt

**Affiliations:** 1 Department of Pathology, The University of Texas Medical Branch, Galveston, Texas, United States of America; 2 Sealy Center for Vaccine Development, The University of Texas Medical Branch, Galveston, Texas, United States of America; 3 Center for Biodefense and Emerging Infectious Diseases, The University of Texas Medical Branch, Galveston, Texas, United States of America; 4 Department of Diagnostic Medicine/Pathobiology, College of Veterinary Medicine, Kansas State University, Manhattan, Kansas, United States of America; 5 Department of Microbiology and Immunology, The University of Texas Medical Branch, Galveston, Texas, United States of America; 6 Galveston National Laboratory, The University of Texas Medical Branch, Galveston, Texas, United States of America; 7 United States Department of Agriculture, Agricultural Research Service, Arthropod Borne Animal Diseases Research Unit, Manhattan, Kansas, United States of America; CDC, UNITED STATES

## Abstract

Rift Valley fever phlebovirus (RVFV) causes high rates of abortions and fetal malformations in ruminants, and hemorrhagic fever, encephalitis, or blindness in humans. Viral transmission occurs via mosquito vectors in endemic areas, which necessitates regular vaccination of susceptible livestock animals to prevent the RVF outbreaks. Although ZH501 strain has been used as a challenge strain for past vaccine efficacy studies, further characterization of other RVFV strains is important to optimize ruminant and nonhuman primate RVFV challenge models. This study aimed to characterize the virulence of wild-type RVFV strains belonging to different genetic lineages in outbred CD1 mice. Mice were intraperitoneally infected with 1x10^3^ PFU of wild-type ZH501, Kenya 9800523, Kenya 90058, Saudi Arabia 200010911, OS1, OS7, SA75, Entebbe, or SA51 strains. Among them, mice infected with SA51, Entebbe, or OS7 strain showed rapid dissemination of virus in livers and peracute necrotic hepatitis at 2–3 dpi. Recombinant SA51 (rSA51) and Zinga (rZinga) strains were recovered by reverse genetics, and their virulence was also tested in CD1 mice. The rSA51 strain reproduced peracute RVF disease in mice, whereas the rZinga strain showed a similar virulence with that of rZH501 strain. This study showed that RVFV strains in different genetic lineages display distinct virulence in outbred mice. Importantly, since wild-type RVFV strains contain defective-interfering RNA or various genetic subpopulations during passage from original viral isolations, recombinant RVFV strains generated by reverse genetics will be better suitable for reproducible challenge studies for vaccine development as well as pathological studies.

## Introduction

Rift Valley fever (RVF) is a mosquito-borne zoonotic viral disease that is endemic to sub-Saharan Africa, but has spread into Egypt, Madagascar, Saudi Arabia, and Yemen. The causative pathogen, Rift Valley fever phlebovirus (RVFV), belongs to the order *Bunyavirales*, family *Phenuiviridae*, genus *Phlebovirus*. RVFV is transovarially transmitted by flood water *Aedes* mosquitoes (e.g., *Ae*. *mcintoshi*, *Ae*. *vexans*), which lay singular eggs above the waterline [1]. These eggs are resistant to draught and can survive for several months; heavy rainfall facilitates the hatching of eggs, resulting in the emergence of infected offspring mosquitoes. Since ruminants in endemic countries are exposed to these mosquitoes, regular vaccination with commercially-available veterinary vaccines is recommended [2, 3]. RVF outbreaks cause devastating damage within the livestock industry due to high rates of abortions and fetal malformations in pregnant ruminants, death of newborn lambs or goat kids, and viral contamination of milk. For example, an RVF outbreak in South Africa in 1951 resulted in the death of 100,000 sheep and abortions in 500,000 ewes [4]. Concurrent with an increase in infected mosquito populations (e.g., *Culex* spp.), humans can be frequently exposed to RVFV. For example, an RVF outbreak in Egypt between 1977 and 1978 resulted in estimated 18,000 to 200,000 human infections and at least 598 deaths [5]. Due to its potential impact on public health upon introduction into non-endemic areas, RVFV is classified as a Category A Priority Pathogen by the National Institute of Health in the United States (U.S.), and as an overlap select agent by the U.S. Department of Agriculture and the U.S. Department of Health and Human Services.

Although there are no clear approaches to eradicating this disease, regular vaccination of susceptible animals is one of the most important mitigation strategies to minimize the spread of RVFV through vector mosquitoes. Importantly, novel candidate RVFV vaccines have the potential to improve the safety and protective efficacy of existing commercial vaccines [3, 6, 7]. There are, however, some limitations in current animal models that likely affect the development of highly potent RVF vaccines: (i) handling of pathogenic RVFV requires high containment facilities, (ii) non-pregnant adult ruminants often show only transient viremia and mild febrile illness, and (iii) genetic variations of pathogenic RVFV strains or stocks likely influence the outcome of vaccine efficacy. It is thus important to understand the differences in virulence among representative RVFV strains and evaluate novel challenge models in ruminants, and other model animals (e.g., mice, nonhuman primates). Since the first reported RVF outbreak in Kenya in 1930–1931 [8], a number of RVFV strains have been isolated throughout endemic countries. A previous study revealed that RVFV strains can be classified into at least seven genetic lineages based on phylogenetic analyses of full-length genome sequences [9]: genetic lineage A (e.g., ZH501), lineage B (e.g., Kenya 199800523, Saudi Arabia 200010911), lineage C (e.g., Zinga), lineage D (e.g., OS1, OS7, SA75), lineage E (e.g., Entebbe), lineage F (e.g., Zimbabwe 2269/74), and lineage G (e.g., SA51). Although RVFV strains differ in nucleotide and amino acid sequences by up to 5% and 2%, respectively [9], it remains unknown whether these strains display distinct virological and pathological phenotypes. One study showed that the median lethal doses (LD50) of ZH501, Entebbe, SA51, and SA75 via subcutaneous infection in adult Wister-Furth rats were 0.7, >6.8, >7.0, and >6.5 Log_10_ plaque forming units (PFU), respectively [10]. This study also showed that Entebbe, SA51, and SA75 strains are more susceptible than ZH501 strain to pre-treatment with rat interferon-α/β. Both ZH501 and SA75 strains were, however, shown to be indistinguishably susceptible to the pretreatment with human IFN-α. Moreover, the Mauritanian RVFV isolates OS1 –OS11 were shown to be highly virulent in suckling mice and adult hamsters, yet those strains were not pathogenic to WF rats [11]. Resistance to rat type-I IFNs does not, therefore, explain the virulence of the RVFV strain in mice, hamsters, humans, or ruminants.

In this study, we characterized the virulence of wild-type (wt) RVFV strains ZH501 (lineage A), Kenya 9800523 (lineage B), Kenya 90058 (lineage B), Saudi Arabia 2000–10911 (lineage B), OS1 (lineage D), OS7 (lineage D), SA75 (lineage D), Entebbe (lineage E), and SA51 (lineage G) using a mouse model. We also generated recombinant SA51 and Zinga strains, both of which are known to be highly pathogenic in adult sheep [12, 13], using a reverse genetics approach.

## Methods

### Media and cells

MRC-5 cells (ATCC CCL-171), OA4.K/S1 cells (ATCC CRL-6549), Vero cells (ATCC CCL-81), and VeroE6 cells (ATCC CRL-1586) were cultured at 37°C with 5% CO_2_ in Dulbecco’s modified minimum essential medium (MEM) containing 10% fetal bovine serum (FBS), penicillin (100 U/ml), and streptomycin (100 μg/ml). BHK/T7-9 cells stably expressing T7 RNA polymerase [14] were cultured at 37°C with 5% CO_2_ in MEM-alpha containing 10% FBS, penicillin (100 U/ml), streptomycin (100 μg/ml), and hygromycin B (600 μg/ml). MRC-5, OA4.K/S1, Vero, VeroE6, and BHK/T7-9 cells used in this study were verified to be mycoplasma free (UTMB Tissue Culture Core Facility), and MRC-5 cells was authenticated by Short Tandem Repeat analysis for the identity (UTMB Molecular Genomics Core Facility).

### Preparation of virus stocks

To characterize the virulence of RVFV strains from different lineages, we prepared RVFV working stocks from those maintained at the University of Texas Medical Branch at Galveston (UTMB). The source and passaging histories of nine wt RVFV strains (ZH501, Kenya 199800523, Kenya 90058, Saudi Arabia 200010911, OS1, OS7, SA75, Entebbe, SA51 strains) were summarized in Table 1. Those strains were further amplified once in Vero cells, and then total RNAs were analyzed by Northern blot to confirm the lack of defective-interfering (DI) RNA, because DI RNA could compromise pathological studies. DI RNAs were indeed detected in the SA75, Entebbe, and SA51 strains (S1 Fig). Plaque cloning was therefore performed for those strains, and DI RNA was eliminated in SA75 clone 4 and SA51 clone 3. Three clones were isolated from parental Entebbe strain stock, yet all the clones still carried DI RNA. The Entebbe strain clone 4, which had only a faint DI RNA band, was thus used for the experiment. Although the other RVFV strains–ZH501, Kenya 199800523, Kenya 90058, Saudi Arabia 200010911, OS1, and OS7 –did not show DI RNA bands in the Northern blot, fast migrating weak bands were additionally detected in RNA from the Kenya 199800523 and Saudi Arabia 200010911 strains. Kenya 199800523 strain clone 3 and Saudi Arabia 200010911 strain clone 1 were therefore isolated and used for the experiments. The sequences of all viral stocks were confirmed by Sanger sequencing (Genewiz, Inc.) (S1 Table).

**Table 1:** Passage histories of wild-type RVFV strain stocks.

Wild-type strains	Source	Year	Country	Passage history	Plaque clones used in this study
ZH501	Human	1997	Egypt	SMB-2, FRhL-2, VeroE6-1, Vero-1	-
Kenya 199800523	Human	1988	Kenya	VeroE6-2	Clone 3 (Vero-2)
Kenya 90058 (B-691)	Bovine	1979	Kenya	Hamster-1, BHK2, FRhL1, VeroE6-1, Vero-1	-
Saudi Arabia 200010911	Human	2000	Saudi Arabia	VeroE6-3	Clone 1 (Vero-2)
OS1	Human	1987	Mauritania	FRhL-1, VeroE6-1, Vero-1	-
OS7	Human	1987	Mauritania	FRhL-1, VeroE6-1, Vero-1	-
SA75	Human	1975	South Africa	FRhL-2, BHK-1, VeroE6	Clone 4 (Vero-2)
Entebbe	Mosquito	1944	Uganda	AM (i.p.)-184, FRhL-1, VeroE6-2	Clone 4 (Vero-2)
SA51	Sheep	1951	South Africa	Sheep-3, SMB-1, FRhL-2, VeroE6-1, Vero-1	Clone 3 (Vero-2)

SMB = suckling mouse brain; FRhL = fetal rhesus lung cell; BHK = baby hamster kidney cell; AM = adult mouse;

i.p. = intraperitoneal;— = plaque purification was not performed.

Recombinant South Africa 1951 (rSA51) strain and recombinant Zinga (rZinga) strain were rescued via reverse genetics, as described previously [15]. Using the TransIT-293 transfection reagent (Mirus Bio), sub-confluent BHK/T7-9 cells were transfected with plasmids expressing full-length L-, M-, or S-segment RNA from either the SA51 or Zinga strains, together with supporting plasmids expressing N, L, and Gn/Gc proteins derived from the ZH501 strain (pT7-IRES-N, pT7-IRES-L, pCAGGS-G) [16]. Culture supernatants were replaced at 1 day post transfection (dpt) and collected at 5 dpt; rescued viruses were then further amplified in Vero cells for 3 to 4 days until extensive cytopathic effects were confirmed. Titers of viral stocks (Vero passage 1: P1) were determined via plaque assay using Vero E6 cells, whereas a lack of DI RNA was tested by Northern blot using total RNA from infected Vero cells (S2 Fig). Stock virus of rSA51 (Vero P1) did not contain detectable DI RNA, whereas stock virus of rZinga (Vero P1) contained a replicating DI RNA. The rZinga strain was thus further plaque-purified, and clone 1 (clear plaques) and clone 3 (turbid plaques) were isolated and amplified twice in Vero cells. Sequences of those stocks were confirmed by Sanger sequencing (Genewiz) (S1 Table).

### Plasmids

The cDNA of the L-, M-, and S-segments of the SA51 or Zinga strains were synthesized (GenScript, Inc.), and then cloned into pProT7 plasmids between the T7 promoter and the hepatitis delta virus ribozyme sequence [15], which generated plasmids expressing full-length antiviral-sense L-, M-, or S-segment RNAs from the SA51 strain (pProT7-SA51-L, pProT7-SA51-M, or pProT7-SA51-S, respectively) or the Zinga strain (pProT7-Zinga-L, pProT7-Zinga-M, or pProT7-Zinga-S, respectively).

### Northern blot analysis

Northern blot analysis with strand-specific RNA probes was performed as described previously [17].

### Mouse experiments

Five-week-old female CD1 mice (Charles River) were intraperitoneally (i.p.) inoculated with 1x10^3^ PFU of recombinant RVFV strains (rZH501, rSA51, or rZinga) or wild-type RVFV strains (ZH501, Kenya 199800523, Kenya 90058, Saudi Arabia 200010911, OS1, OS7, SA75, Entebbe, or SA51) (n = 10 per group). Clinical scoring and health assessments were monitored daily until 21 days post infection (dpi) by visual examination, and twice daily, once they reached a total clinical scoring of 4–7 within the health evaluation criteria, which indicates evidence of pain or discomfort, based on appearance (score range 0–3), body weight (score range 0–3), unprovoked behavior (score range 0–3), clinical signs (score range 0–3), and behavior response to external stimuli (score range 0–2). Mice with clinical signs of severe diseases, such as body weight loss of more than 20%, paralysis, or lethargy, were humanely euthanized, whereas all surviving mice were euthanized at 21 dpi. Mice were euthanized by an inhalation of 2–5% isoflurane to effect via vaporizer, followed by cervical dislocation. Livers and spleens of selected sick mice were fixed in 10% buffered formalin for postmortem histopathological analysis. Mice were also i.p. inoculated with 1x10^0^, 1x10^1^, 1x10^2^, or 1x10^3^ PFU of rZH501, rSA51, or rZinga strain, which were not plaque-cloned, and the survivals were monitored for 21 dpi. The LD50 was calculated using the Reed and Muench method [18].

### Histopathological examinations

Livers and spleens were fixed with 10% neutral buffered formalin and embedded in paraffin blocks, and then thin sections were stained with hematoxylin-eosin staining (H.E.). For immunohistochemistry (IHC), sectioned tissues were treated with proteinase K (Dako, Carpinteria, CA), followed by anti-RVFV N rabbit polyclonal antibody [19] at a dilution of 1 in 500 and biotinylated secondary goat anti-rabbit IgG antibody (Vector Laboratories, Burlingame, CA) at a dilution of 1 in 200. Sections were further incubated with streptavidin alkaline phosphatase (Vector Laboratories) and then the Fast Red substrate (Dako), before being additionally stained with hematoxylin to visualize the background. Uninfected mouse tissue sections were used as a negative control for primary antibodies. Images were captured via DP Manager software using a DP71 camera attached to an Olympus IX71 microscope. Color deconvolution of captured TIFF images was subsequently performed via FIJI Image J software [20, 21] using the H&E channel (Image 1: Red: 0.64, Green: 0.72, Blue: 0.27; Image 2: Red: 0.092, Green: 0.95, Blue: 0.28; Image 3: Red: 0.64, Green 0.001, Blue 0.77). Image 2, which reflected red color without the hematoxylin background, was used for analysis. The threshold of Image 2 was further adjusted to reflect the intensity of positive signals, and the converted black and white image was used for the measurement of area. The area of the image was measured with and without the threshold, and the percentage of the area of positive IHC signals normalized to the entire tissue area was calculated.

### Statistical analysis

GraphPad Prism 6.05 (GraphPad Software Inc.) was used for Kaplan-Meier survival curve analysis via Log-rank test. To compare virus titers among multiple groups, arithmetic means of log_10_ values were analyzed by one-way ANOVA, followed by Tukey’s multiple comparison test.

### Ethics statement

Usage of recombinant DNA or biologicals was approved by the Institutional Biosafety Committee at UTMB. All *in vitro* experiments and mouse studies with wt RVFV (select agent) were performed in the UTMB Robert E. Shope BSL-4 laboratory or Galveston National Laboratory, accredited by the Association for Assessment and Accreditation of Laboratory Animal Care in accordance with the Animal Welfare Act, NIH guidelines, and U.S. federal law. Animal protocols were approved by the UTMB Institutional Animal Care and Use Committee (protocol number 1412066).

## Results

### Virulence of wild-type RVFV strains from distinct genetic lineages in outbred mice

**Fig 1:**
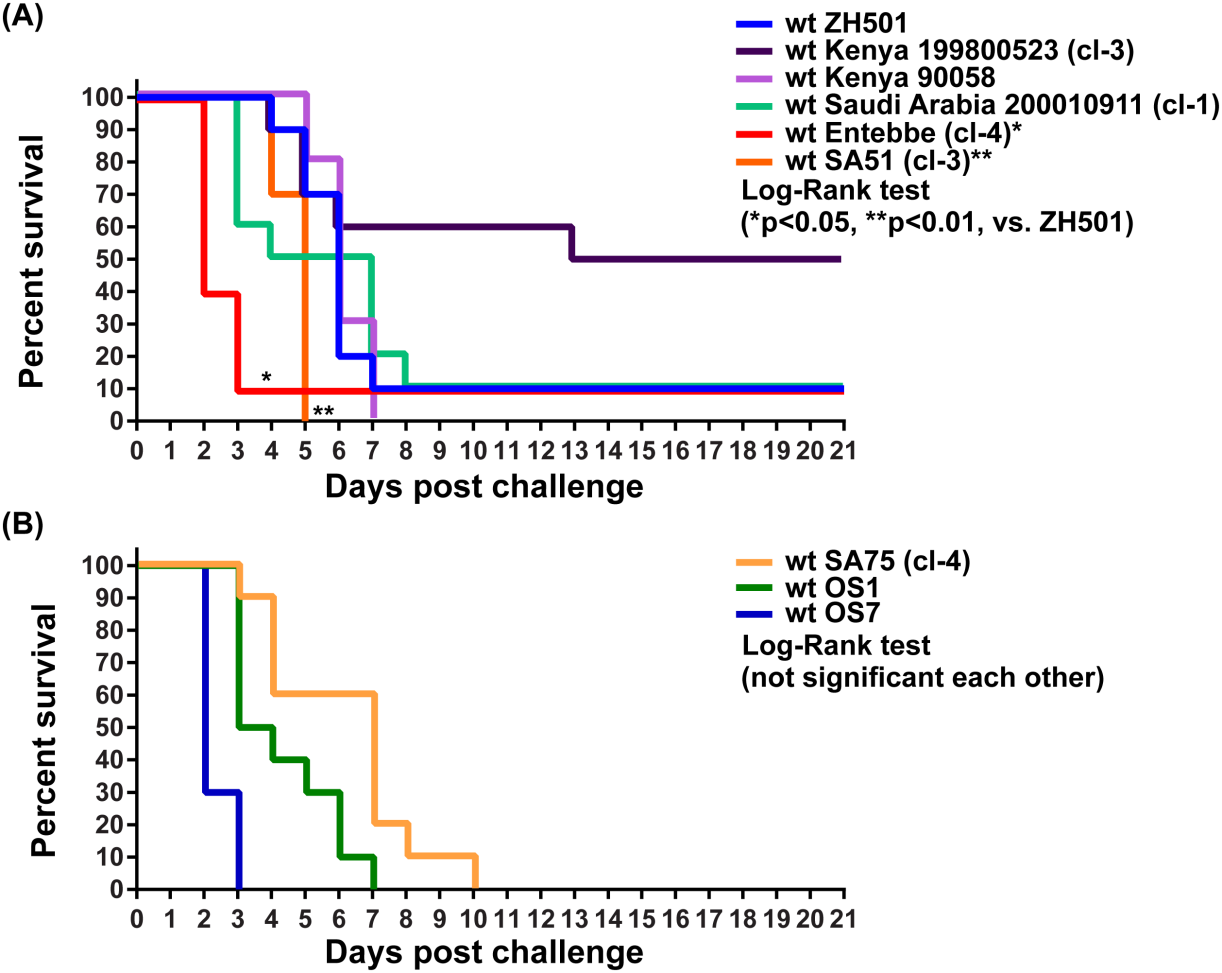
Virulence of wild-type (wt) Rift Valley fever phlebovirus (RVFV) strains in mice. Five-week-old outbred CD1 mice were intraperitoneally inoculated with 1x10^3^ PFU of nine different wt RVFV strains: (A) ZH501, Kenya 199800523, Kenya 90058, Saudi Arabia 200010911, Entebbe, or SA51; (B) OS1, OS7, or SA75. The Kaplan-Meier survival curves (n = 10 per group) of infected mice are shown. Asterisks represent the statistically significant differences based on log-rank testing (*p < 0.05, **p < 0.01, vs. ZH501).

We performed a mouse challenge study to analyze the virulence of different RVFV strains. Pathogenic wt RVFV strains (ZH501, Kenya 199800523, Kenya 90058, Saudi Arabia 200010911, OS1, OS7, SA75, Entebbe, and SA51) were amplified in Vero cells to prepare working stocks of those strains (Table 1). To eliminate DI RNAs, the Kenya 199800523, Saudi Arabia 200010911, SA75, Entebbe, and SA51 strains were further plaque cloned (S1 Fig). Five-week-old outbred CD1 mice were inoculated (i.p.) with 1x10^3^ PFU of wt RVFV strains ZH501, Kenya 199800523, Kenya 90058, Saudi Arabia 200010911, OS1, OS7, SA75, Entebbe, or SA51. Retitration of inocula determined actual viral doses as follows: 30%, 42%, 90%, 40%, 300%, 400%, 400%, 30%, or 50%, respectively, relative to the target dose 1x10^3^ PFU (100%) (S2 Fig). Kaplan Meier survival curves of lower dose mice groups (ZH501, Kenya 199800523, Kenya 90058, Saudi Arabia 200010911, Entebbe, or SA51) and higher dose groups (SA75, OS1, or OS7) were thus separately shown (Fig 1). Survival rates of infected mice were 10% (ZH501), 50% (Kenya 199800523), 0% (Kenya 90058), 10% (Saudi 200010911), 10% (Entebbe), 0% (SA51) (Fig 1A), 0% (OS1), 0% (OS7), and 0% (SA75) (Fig 1B).

Log-rank testing showed that the survival curves of mice infected with the Entebbe or SA51 strains were significantly different from that of ZH501-infected mice. The median survival times of infected mice were 6.0 (ZH501), 6.0 (Kenya 90058), 17.0 (Kenya 199800523), 5.5 (Saudi Arabia 200010911), 2.0 (Entebbe), 5.0 (SA51), 3.5 (OS1), 2.0 (OS7), or 7.0 days (SA75). Although one mouse survived infection with the Entebbe strain, the other nine mice were all found dead at 2 dpi, before exhibiting any clinical signs of disease (S1 Fig). Similar peracute onset of disease also occurred in 70% of OS7-infected mice, and in 30% of Saudi 200010911-infected mice. Overall, results indicated that the virulence of at least wt SA51, Entebbe, and OS7 strains in mice could be distinct from that of the ZH501 strain.

### Characterization of recombinant SA51 strain and Zinga strain

**Fig 2:**
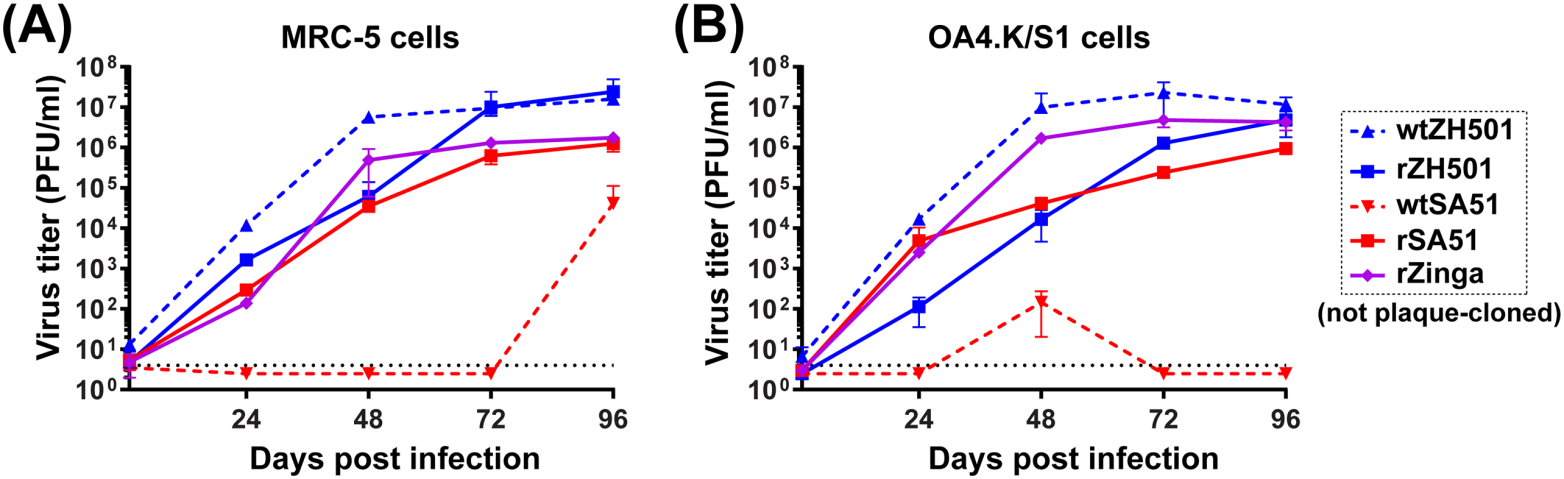
Replication kinetics of recombinant South Africa 1951 (rSA51) and Zinga (rZinga) strains in culture cells. (A–B) Replication kinetics of recombinant ZH501 (rZH501), rSA51, and rZinga strains or wt ZH501 or wt SA51 strains in human lung diploid MRC-5 cells (A) or sheep kidney OA4.K/S1 cells (B). Cells were infected with RVFV at MOI 0.01. Infectious virus titers (plaque-forming units, PFU) were determined by plaque assay.

Although wt RVFV strains showed distinct virulence in mice, variations in the passage history of stock viruses and the presence of DI RNAs could alter virulence in animals. To allow reproducible animal challenge experiments, we developed a reverse genetics system for the highly-pathogenic SA51 strain, the most distant strain from well-characterized ZH501 strain. In addition, a reverse genetics system for the Zinga strain was also developed to analyze the virulence of a RVFV strain belonging to the genetic lineage C. Recombinant RVFV strains rSA51 and rZinga were rescued from synthesized cDNA using reverse genetics, as described previously [15, 22]. Both recombinant strains were viable and were amplified in Vero cells. Although the consensus sequences of rSA51 and rZinga stocks were identical to those of the reference sequences, the rZinga strain stock, but not rZH501 or rSA51 stocks, contained DI RNA after an amplification in Vero cells (S3 Fig). Next, parental stocks of rSA51, rZinga, and rZH501, which were not plaque-cloned, were used for the analysis of virus replication kinetics in MRC-5 cells and OA4.K/S1 cells at MOI 0.01 (Fig 2). For the comparison, wt SA51 strain and wt ZH501 strain, both of which were not plaque-cloned, were also included in the experiment. Both, the rSA51 and rZinga strains replicated in those cells as efficiently as did the recombinant ZH501 (rZH501) strain or wt ZH501 strain. It was noted that wt SA51 strain, which contains detectable DI RNA (S1 Fig), failed to replicate in MRC-5 or OA4.K/S1 cells.

**Fig 3:**
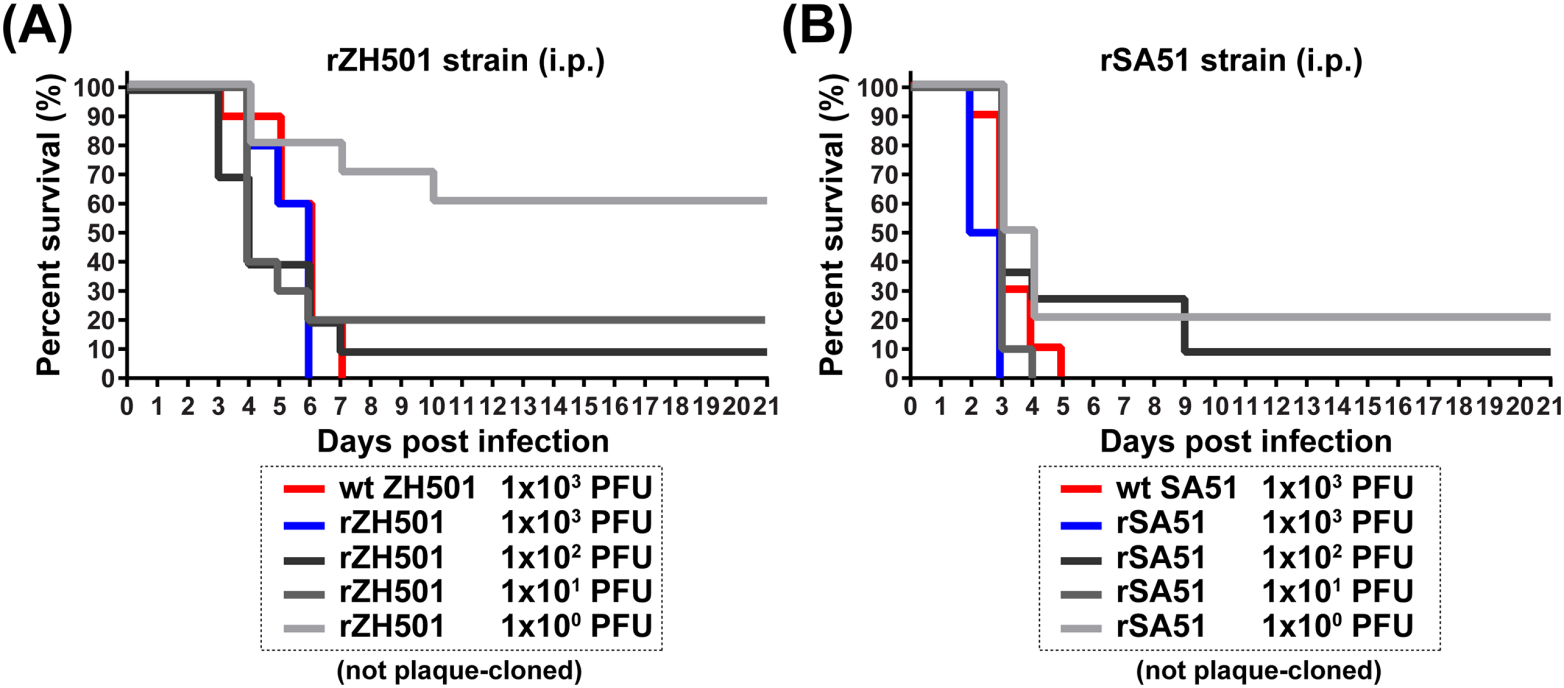
Virulence of recombinant South Africa 1951 (rSA51) and rZH501 strains in mice. Five-week-old outbred female mice (n = 10 per group) were intraperitoneally inoculated with 1x10^0^, 1x10^1^, 1x10^2^, or 1x10^3^ PFU of rZH501 or rSA51 strain, or 1x10^3^ PFU of wt ZH501 or wt SA51 strains. The Kaplan-Meier survival curves of mice infected with the rZH501 or wt ZH501 strain (A) or rSA51 or wt SA51 strain (B) are shown.

Next, five-week-old outbred CD1 mice were inoculated (i.p.) with different doses (1x10^0^, 1x10^1^, 1x10^2^, or 1x10^3^ PFU) of rZH501 or rSA51 strains, or 1x10^3^ PFU of wt ZH501 or wt SA51 strains (Fig 3). In mice inoculated with the rZH501 strain, survival rates of mice inoculated with 1x10^0^, 1x10^1^, 1x10^2^, or 1x10^3^ PFU were 60%, 20%, 10%, and 0%, respectively (LD50 = 5.6 PFU). Survival rates of mice inoculated with the rSA51 strain were 20%, 0%, 10%, and 0% for 1x10^0^, 1x10^1^, 1x10^2^, and 1x10^3^ PFU doses, respectively (LD50 ≤ 1 PFU). The median survival times of mice inoculated with 1x10^3^ PFU of rZH501 or rSA51 were 6 and 2.5 days, respectively. Log-rank testing showed that the survival curve of rSA51-infected mice was significantly different from that of rZH501-infected mice (1x10^3^ PFU dose: p < 0.01; 1x10^0^ PFU dose: p < 0.05; 1x10^1^ and 1x10^2^ PFU doses: not significant). Overall, mice infected with rSA51 showed a faster onset of disease even at lower doses (S4 Fig) showing that the rSA51 strain is more virulent in mice than are the rZH501 strain.

**Fig 4:**
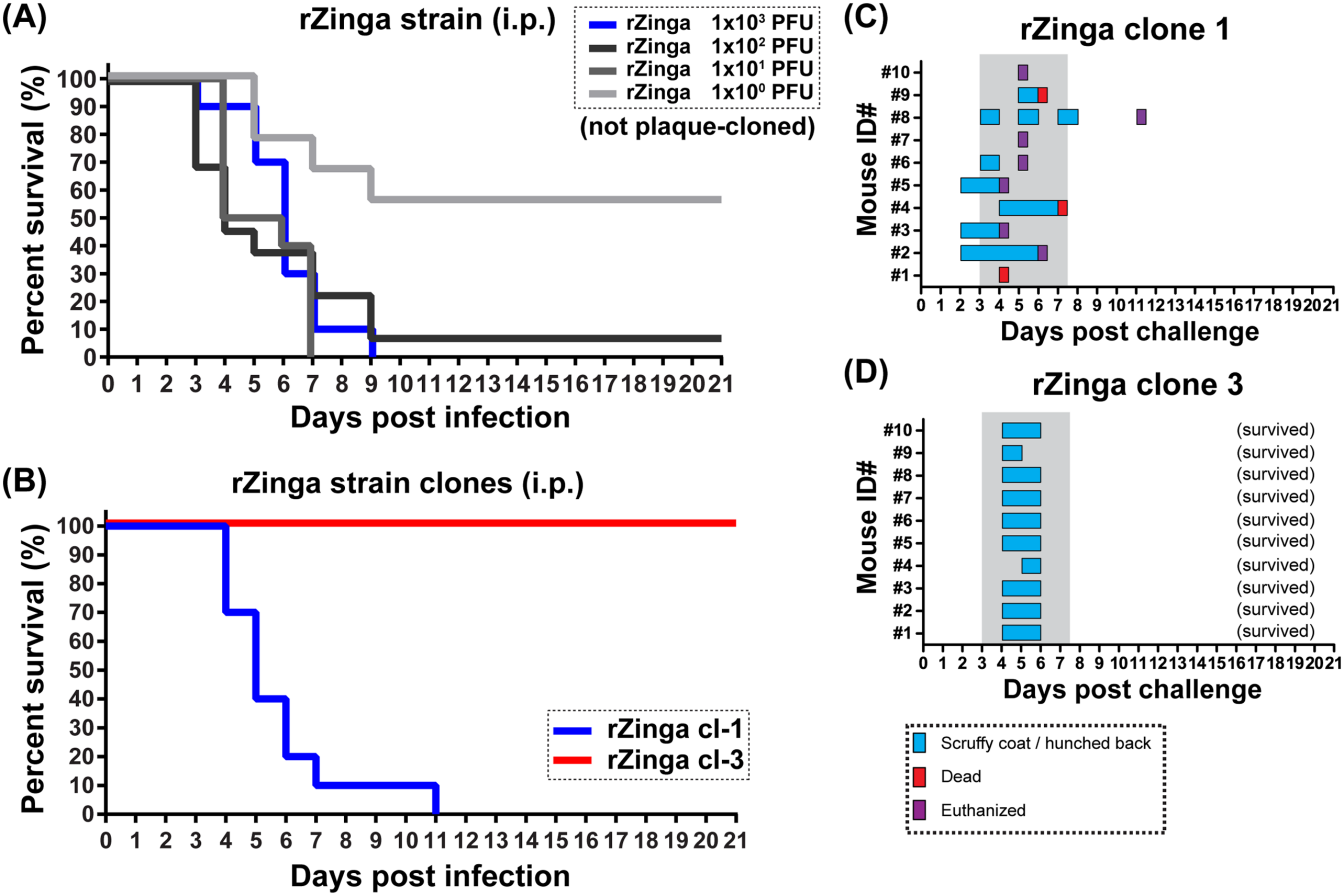
Distinct phenotypes of recombinant Zinga (rZinga) strain plaque clones in mice. (A) Five-week-old outbred female mice (n = 10 per group) were intraperitoneally inoculated with 1x10^0^, 1x10^1^, 1x10^2^, or 1x10^3^ PFU of rZinga strain, which was not plaque-cloned. The Kaplan-Meier survival curves are shown. (B) The Kaplan-Meier survival curves of mice intraperitoneally infected with 1x10^3^ PFU of rZinga strain clone 1 or clone 3. (C–D) Clinical signs of disease observed in each mouse are shown: (C) rZinga clone 1 and (D) rZinga clone 3. Blue = scruffy coat and/or hunched back; red = dead; purple = euthanized.

Similarly, mice were inoculated (i.p.) with different doses (1x10^0^, 1x10^1^, 1x10^2^, or 1x10^3^ PFU) of rZinga strain, which was not plaque-cloned (Fig 4A). Survival rates of mice inoculated with the rZinga strain at 1x10^0^, 1x10^1^, 1x10^2^, or 1x10^3^ PFU were 60%, 0%, 10%, and 0%, respectively (LD50 = 10 PFU). The median survival times of mice inoculated with 1x10^3^ PFU of rZinga strain was 6 days, and the survival curve of rZinga-infected mice was not significantly different from that of rZH501-infected mice at either of 1x10^0^, 1x10^1^, 1x10^2^, or 1x10^3^ PFU by the Log-rank test. Since the rZinga strain contained DI RNA in viral stock (S3 Fig), the virulence of plaque clone 1 and 3 of the rZinga strain was further analyzed in mice, which were no longer contained detectable DI RNAs. Mice infected with rZinga clone 1 showed a survival curve similar to that of the parental rZinga strain, with median survival times of 5.0 days (Fig 4B and 4C). In contrast, all mice infected with rZinga clone 3 survived the challenge, with transient clinical signs of disease manifesting at 4 or 5 dpi (Fig 4B and 4D). The rZinga clone 1 encoded E216G mutation in the N gene, whereas the rZinga clone 3 encoded L218R mutation in the NSs gene and E361G mutation in the L gene (S1 Table). The plaque phenotype was similar between parental rZinga (uncloned) and the clone 1, whereas the clone 3 formed turbid plaques in Vero E6 cells in a plaque assay.

### Characterization of histopathological changes at 2 dpi

**Fig 5:**
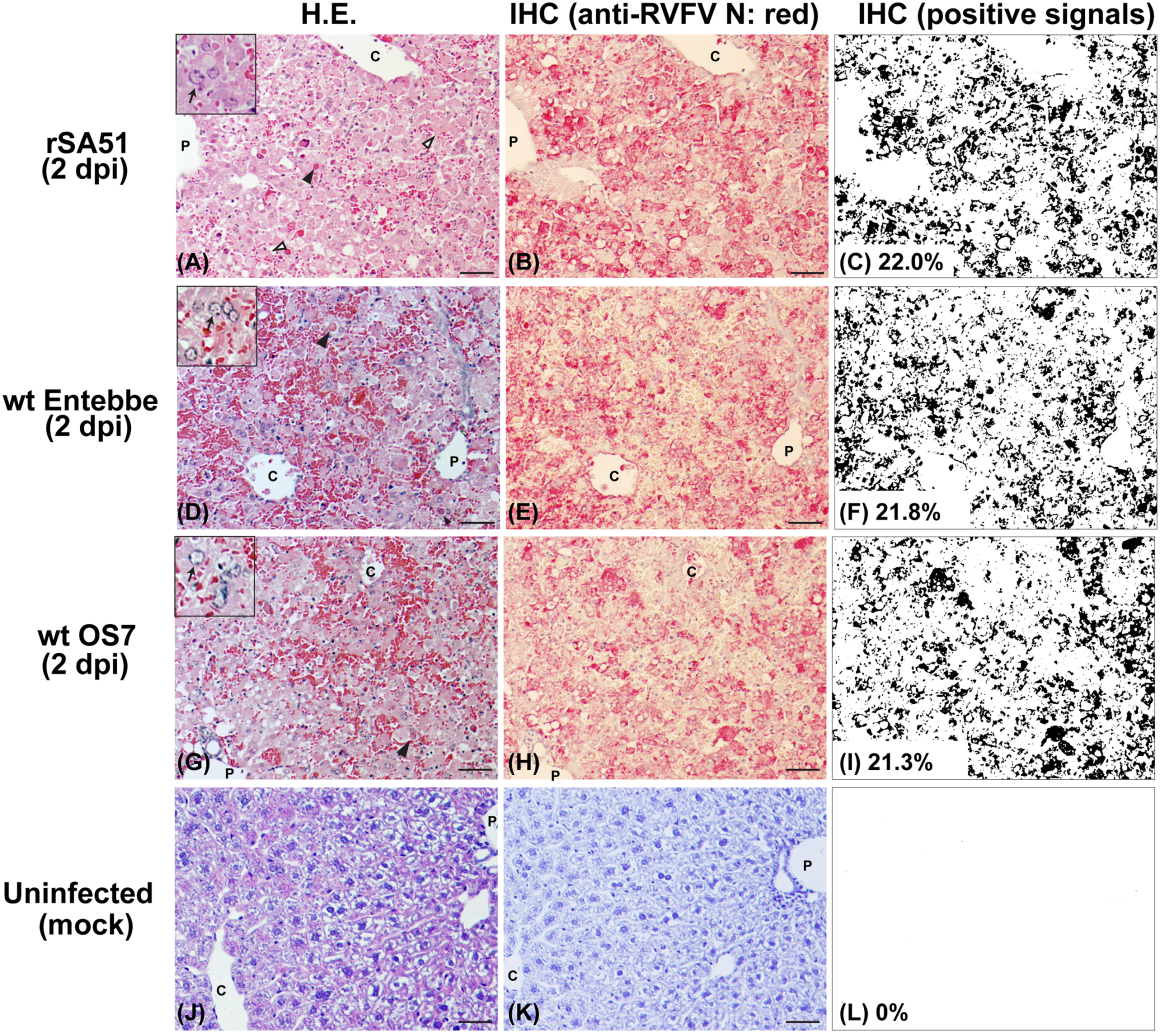
Histopathological changes in livers of mice infected with the rSA51, wt Entebbe, or wt OS7 strains of Rift Valley fever phlebovirus (RVFV) at 2 days post infection (dpi). Mice intraperitoneally infected with 1x10^3^ PFU of rSA51 (A–C), wt Entebbe clone 4 (D–F), or wt OS7 (G–I) were euthanized at 2 dpi. A mouse intraperitoneally mock-infected with PBS (J–L) was used as a control. Liver tissues, which were fixed with 10% neutral buffered formalin and embedded in paraffin blocks, were used for histopathological examinations. Thin sections were stained with hematoxylin-eosin staining (A, D, G, and J). Corresponding lesions were also incubated with anti-RVFV N rabbit polyclonal antibody to analyze immunohistochemistry (IHC; B, E, H, and K). Specific signals (red) derived from Vector Red Alkaline Phosphatase substrate were detected via the streptavidin-biotin method using the UltraVision Alk-Phos kit. Quantification of the area of positive signals normalized to the tissue area in each IHC image was performed via FIJI Image J software after color deconvolution (C, F, I, and L). Arrowheads (A, D, and G) indicate Councilman bodies, and inset images (A, D, and G) show hepatocytes with filamentous eosinophilic intranuclear inclusion bodies (arrows). C = central vein; P = portal vein. Bars represent 50 μm.

**Fig 6:**
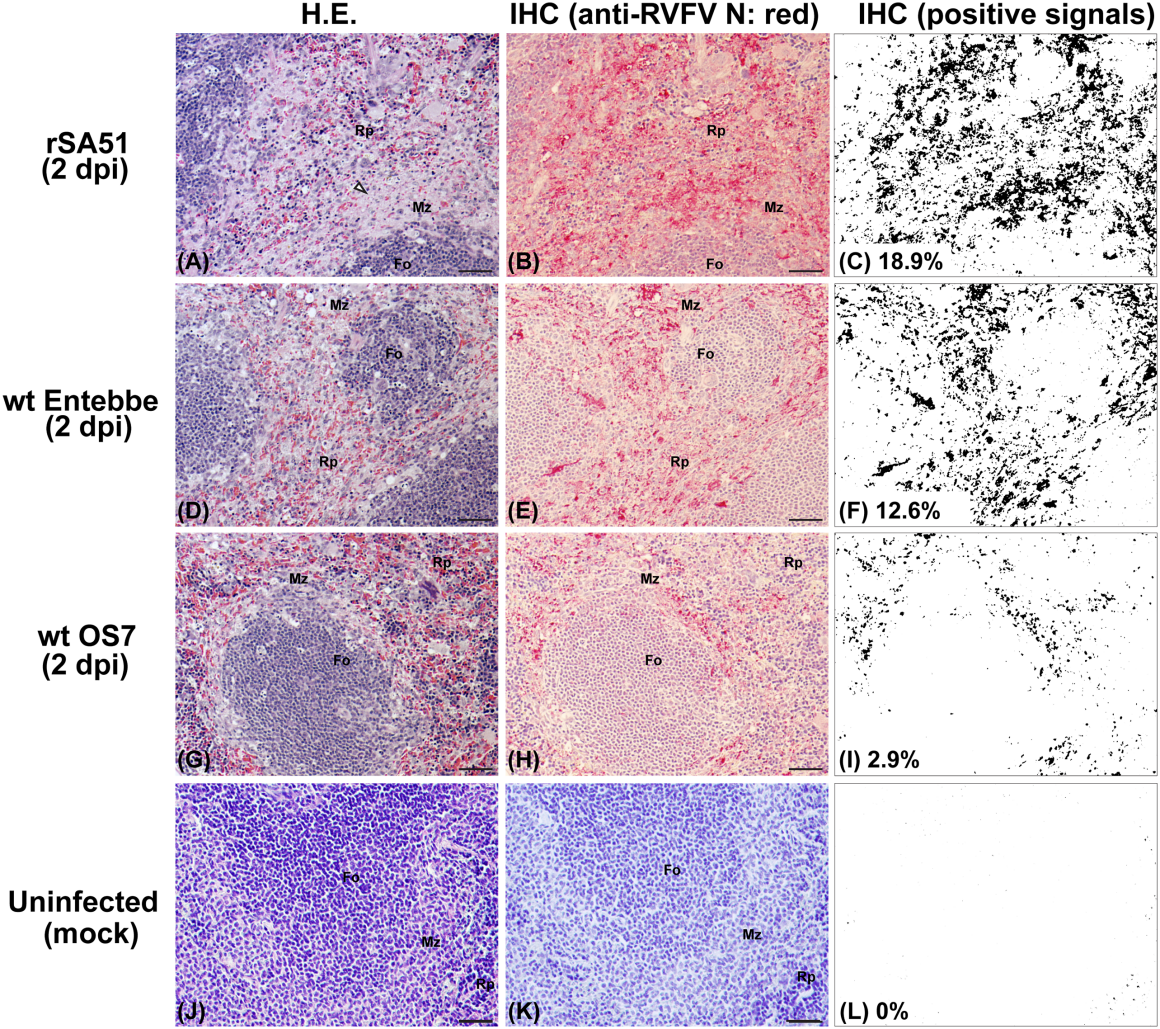
Histopathological changes of spleens in mice infected with rSA51, wt Entebbe, or wt OS7 strains of Rift Valley fever phlebovirus (RVFV) at 2 days post infection (dpi). Mice intraperitoneally infected with 1x10^3^ PFU of rSA51 (A–C), wt Entebbe (D–F), or wt OS7 (G–I) were euthanized at 2 dpi. A mouse intraperitoneally mock-infected with PBS (J–L) was used as a control. Spleen sections were stained with hematoxylin-eosin staining (A, D, G, and J). Corresponding lesions were also incubated with anti-RVFV N rabbit polyclonal antibody to analyze immunohistochemistry (IHC; B, E, H, and K). Quantification of the area of positive signals normalized to the tissue area in each IHC image is also shown (C, F, I, and L). Arrowheads (A) indicate necrotic area in the marginal zone. Rp = red pulp; Fo = follicle; Mz = marginal zone. Bars represent 50 μm.

To further understand peracute RVF disease that occurred at 2 dpi in mice infected with rSA51, wt Entebbe, or wt OS7, livers and spleens were histopathologically examined. IHC signals were analyzed via FIJI Image J software after color deconvolution. The reactivity of anti-RVFV N rabbit antibody was validated using mock-infected mouse tissues. Although mice were euthanized at 2 dpi, viral antigens were abundantly detected in livers or spleen from those infected with rSA51 (IHC positive area: liver 22.0%, spleen 18.9%), Entebbe (IHC positive area: liver 21.8%, spleen 12.6%), or OS7 (IHC positive area: liver 21.3%, spleen 2.9%) (Figs 5 and 6). Hepatocytes were necrotic in most areas of the liver lobule, and Councilman bodies were readily detected (Fig 5A, 5D and 5G). Filamentous eosinophilic intranuclear inclusion bodies were also sporadically found in hepatocytes. Infiltration of inflammatory cell was not found in portal triads and liver parenchyma, whereas red blood cells accumulated between necrotic hepatocytes. In spleens, viral antigens were detected in mononuclear cells in marginal zones and red pulp, indicating the infection of macrophages, dendritic cells, and/or reticular cells (Fig 6). H.E. staining showed that in rSA51-infected mice, cells in the marginal zone of the spleen were necrotic.

Histopathological analyses were also performed for liver and spleen tissues infected with wt OS1 (3 dpi), wt Saudi Arabia 20010911 (3 dpi), rZinga clone 1 (4 dpi), wt Kenya 199800523 (4 dpi), wt ZH501 (4 dpi), or wt SA75 (4 dpi) (S5 and S6 Figs). Coagulative necrosis of hepatocytes was sporadically or diffusely detected in those liver sections, and hepatocytes with ballooning degeneration were also present. In spleens, mononuclear cells with viral antigens were scattered in the marginal zone and red pulp.

## Discussion

Wild-type RVFV strains have been isolated and passaged using various methods, making it likely for each viral stock to have unique genetic subpopulations. Genetic diversity most likely will affect virulence [23], and DI RNA potentially interferes with the replication of pathogenic strains via direct competition for viral proteins or induction of host innate immunity [24–26]. The evaluation of virological and pathological phenotypes using stock viruses was thus challenging. We initiated the analysis of DI RNA in each viral stock and found that the wt Entebbe, wt SA51, and wt SA75 strain stocks indeed contained detectable DI RNA. Those DI RNA were eliminated from wt SA51 and wt SA75 stocks after plaque cloning, whereas DI RNA remained detectable in all four plaque-purified isolates from the wt Entebbe strain stock. All clones of RVFV stocks were further sequenced before the mouse experiment, resulting in the discovery of several variations in the sequence in comparison to reported consensus sequences. It is therefore not clear how the selection of specific genetic populations affected the outcome of the mouse survival study. The only effective approach to address this issue was to establish reverse genetic systems for the individual viruses.

The mortality rate of adult sheep (7 months– 5 years old) caused by the SA51 strain has been reported to be 20% [12], whereas the mortality rate of adult sheep (7–11 months old, Yansaka, Ouda, or WAD breed) by the Zinga strain has been reported to be 100% in each breed [13]. In addition to the currently-available rZH501 strain, the rSA51 and rZinga strains could be useful additions for studying pathogenesis and vaccine efficacy in ruminants. We thus developed two reverse genetics systems to rescue the rSA51 and rZinga strains. Both of them replicated efficiently in MRC-5 or OA4.K/S1 cells, although uncloned wt SA51 strain failed to replicate in those cells, probably due to the presence of DI RNAs. The median survival times of infected mice were 3 days for wt SA51 (uncloned), 5 days for wt SA51 clone 3, and 2.5 days for rSA51 (1x10^3^ PFU). The rSA51 strain stock did not contain detectable DI RNA and viral sequence did not encode amino acid substitutions compared to the parental wt SA51 strain sequence. In contrast, the wt SA51 plaque clone (clone 3) encoded three amino acid substitutions (Gn: N252D, L: N284S, L: N476H). The reverse genetics for SA51 strain will be thus useful to reproduce high virulence in mice with less concerns for DI RNA or amino acid mutations. The virulence of rZinga strain without plaque-cloning was similar to that of rZH501 strain. We noted, however, the presence of DI RNA in the rZinga strain stock, which experienced one passage in Vero cells after the recovery in BHK/T7-9 cells. Two plaque-clones (clone 1 and 3) did not contain detectable DI RNA, yet showed different virulence in mice. The rZinga strain clone 3, which encodes the L218R mutation in the NSs gene and E361G mutation in the L gene, was not pathogenic to mice, indicating that either or both of the mutations could affect the virulence of RVFV in mice. The result indicates that the plaque cloning and the quality validation of viral RNA (i.e., lacks of mutations or DI RNA) is important to prepare viral stocks for recombinant RVFV strains via reverse genetics.

Peracute onset of RVF disease occurs in newborn lambs, goat kids, hamsters, specific inbred rat strains (e.g., WF or Brown Norway) and inbred mouse strains (e.g., MBT/Pas) [4, 27–29]. Host factors (e.g., age, genetic background, species) are thus important determinants of susceptibility to RVFV. Our study showed that at least three (SA51, Entebbe, and OS7 strains) out of nine RVFV strains induce peracute liver necrosis in five-week-old outbred CD1 mice, which have diverse genetic backgrounds. This shows that viral dissemination in mice early in infection is influenced by the strains of RVFV. Mice euthanized with peracute disease at 2 dpi showed diffuse necrosis of hepatocytes, as well as diffuse viral spread in liver and spleen. Councilman bodies, which represent dying hepatocytes, were occasionally found in liver parenchyma, indicating that hepatocytes undergo apoptosis at 2 dpi. In spleens, viral antigens were frequently detected in mononuclear cells in marginal zones, among different challenge groups. It is important to note that marginal zone macrophages (MGMs) are responsible for controlling systemic viral spread during the acute phase in lymphocytic choriomeningitis virus infection in mice [30]. Early infection and dysregulation of MGMs by RVFV may affect the physiological function of these cells facilitating systemic viral spread via infected blood cells. The viral determinant of rapid proliferation of RVFV in liver and spleen, however, requires further study.

Although amino acid differences in Gn/Gc proteins among RVFV strains can reach 2%, few studies have shown the cross-neutralization of different RVFV strains via antisera from vaccinated animals. Battles et al. showed that neutralizing monoclonal antibodies 4D4 and 4-39-CC, which recognize epitope II (amino acids 382–392 in the Gn) and epitope IV (amino acids 280–299 in the Gn), respectively, results in the cross-neutralizing activity of several RVFV strains, including ZH501, Entebbe, Zinga, SA51, and SA75 [31]. Reduced neutralization of the Smithburn neurotropic strain was, however, reported by two different research groups [31, 32]. We additionally analyzed the cross-neutralization activities of antisera derived from sheep or cattle vaccinated with either MP-12 or rMP12-ΔNSm21/384 (S7 Fig) [7]. Four sera derived from ewes vaccinated with either MP-12 or rMP12-ΔNSm21/384, and two cattle sera vaccinated with rMP12-ΔNSm21/384, were subjected to a plaque reduction neutralization test using six different RVFV strains: MP-12, wt Kenya 199800523, rZinga, wt OS1, wt Entebbe, and wt SA51. All six sera successfully reduced the formation of plaques for all tested RVFV strains by at least 80% using a 1:160 serum dilution, indicating that vaccination against the MP-12 strain can induce antibodies cross-neutralizing RVFV strains belonging to different genetic lineages. Due to the presence of indistinguishable cross-neutralizing activity among different RVFV strains, a selection of specific strain will not increase a bias in the protective efficacy of vaccine candidates using a RVFV challenge model.

## Conclusions

RVFV strains belonging to different genetic lineages show distinct virulence in outbred mice, although those strains are antigenically similar. Since wt RVFV strains might contain DI RNA or different genetic subpopulations during the passage from original viral isolations, recombinant RVFV strains such as rSA51 and rZinga, generated by reverse genetics, will be more suitable for reproducible challenge studies for vaccine development.

## Supporting information

S1 Fig Northern blot analysis of wild-type (wt) Rift Valley fever phlebovirus stocks. Total RNA was extracted from Vero cells infected with each stock virus (parental stock, Vero P1 of parental stock, or Vero P2 of plaque clones) at 24 hours post infection. Northern blot used RNA probes specific to antiviral-sense (+) or viral-sense (-) L, M-, or S-segment RNA. (A) wt ZH501, (B) wt Kenya 199800523, (C) wt Kenya 90058, (D) wt Saudi Arabia 200010911, (E) wt OS1, (F) wt OS7, (G) wt SA75, (H) wt Entebbe, (I) wt SA51. Defective-interfering RNA is shown in red. Probes (-) or Probes (+) represent a mixture of RNA probes detecting negative-sense or positive-sense L-, M-, and S-segments, respectively.
(TIF)

S2 Fig Virulence analysis of wild-type (wt) Rift Valley fever phlebovirus (RVFV) strains in mice. (A) Actual viral doses of wt RVFV strains; ZH501, Kenya 199800523, Kenya 90058, Saudi Arabia 200010911, OS1, OS7, SA75, Entebbe, or SA51. Percentages to the target dose (1x10^3^ PFU) are also shown. (B–K) Clinical signs of disease observed in each mouse are shown in relation to viral infection: (B) wt ZH501, (C) wt Kenya 199800523 cl-3, (D) wt Kenya 90058, (E) wt Saudi Arabia 200010911 cl-1, (F) wt OS1, (G) wt OS7, (H) wt SA75 cl-4, (I) wt Entebbe cl-4, and (J) wt SA51 cl-3. Blue = scruffy coat and/or hunched back; red = dead; purple = euthanized.
(TIF)

S3 Fig Northern blot analysis of recombinant Rift Valley fever phlebovirus stocks. Total RNA was extracted from Vero cells infected with each stock virus (Vero P1 of rescued P0 virus or Vero P2 of plaque clones) at 24 hours post infection. Northern blot used RNA probes specific to antiviral-sense (+) or viral-sense (-) L, M-, or S-segment RNA. (A) recombinant ZH501 (rZH501), (B) recombinant SA51 (rSA51), (C) recombinant Zinga (rZinga). Defective-interfering RNA is shown in red. Probes (-) or Probes (+) represent a mixture of RNA probes detecting negative-sense or positive-sense L-, M-, and S-segments, respectively.
(TIF)

S4 Fig Clinical signs of disease observed in mice infected with rZH501, rSA51, or rZinga strains. Clinical signs of disease observed in each mouse are shown in each group: (A) rZH501, 1x10^0^ PFU, (B) rZH501, 1x10^1^ PFU, (C) rZH501, 1x10^2^ PFU, (D) rSA51, 1x10^0^ PFU, (E) rSA51, 1x10^1^ PFU, (F) rSA51, 1x10^2^ PFU, (G) rZinga, 1x10^0^ PFU, (H) rZinga, 1x10^1^ PFU, and (I) rZinga, 1x10^2^ PFU. Blue = scruffy coat and/or hunched back; red = dead; purple = euthanized.
(TIF)

S5 Fig Histopathological changes in livers of mice infected with pathogenic Rift Valley fever phlebovirus (RVFV) strains at 3 or 4 days post infection. Livers of mice intraperitoneally infected with 1x10^3^ PFU of wt OS1 (A–C), wt Saudi Arabia 20010911 (D–F), rZinga (G–I), wt Kenya 199800523 (J–L), wt ZH501 (M–O), or wt SA75 (P–R) were histopathologically analyzed via hematoxylin-eosin staining (A, D, G, J, M, and P) or immunohistochemistry (IHC) using anti-RVFV N rabbit polyclonal antibody (B, E, H, K, N, and Q). The percentage of the area of positive signals was shown in each IHC image: entire the tissue area was set as 100% (C, F, I, L, O, and R). C = central vein; P = portal vein. Bars represent 50 μm.
(TIF)

S6 Fig Histopathological changes in spleens of mice infected with pathogenic Rift Valley fever phlebovirus (RVFV) strains at 3 or 4 days post infection. Spleens of mice intraperitoneally infected with 1x10^3^ PFU of wt OS1 (A–C), wt Saudi Arabia 20010911 (D–F), rZinga (G–I), wt Kenya 199800523 (J–L), wt ZH501 (M–O), or wt SA75 (P–R) were histopathologically analyzed via hematoxylin-eosin staining (A, D, G, J, M, and P) or immunohistochemistry (IHC) using anti-RVFV N rabbit polyclonal antibody (B, E, H, K, N, and Q). The percentage of the area of positive signals was shown in each IHC image: entire the tissue area was set as 100% (C, F, I, L, O, and R). Rp = red pulp; Fo = follicle; Mz = marginal zone. Bars represent 50 μm.
(TIF)

S7 Fig Cross-neutralization of Rift Valley fever phlebovirus strains by sheep or cattle sera vaccinated with MP-12 or rMP12-ΔNSm21/384. Amino acid sequences of Gn (A) or Gc (B) proteins were compared among RVFV strains MP-12 (GenBank accession DQ380208), ZH501 (DQ380200), Kenya 9800523 (DQ380196), Saudi Arabia 200010911 (DQ380197), Zinga (DQ380217), OS1 (DQ380186), SA75 (DQ380189), Entebbe (DQ380191), and SA51 (DQ380195). Amino acid positions are shown based on the precursor protein from the 1^st^ AUG start codon in the M-segment. Locations of neutralizing epitopes I, II, and IV [33] are also shown. TMD, transmembrane domain; CT, cytoplasmic tail. (B) Serially four-fold diluted sera derived from pregnant ewes or cattle vaccinated with MP-12 or rMP12-ΔNSm21/384 [34, 35], were incubated with approximately 50 PFU of MP-12, wt Kenya 199800523, rZinga, wt OS1, wt Entebbe, or wt SA51 for the PRNT_80_. The reduction % of plaque numbers was calculated: the plaque number with a normal control serum was set as 100%.
(TIF)

S1 Table Sequence changes of wild-type Rift Valley fever phlebovirus (wt RVFV) and recombinant RVFV (rRVFV) stocks in comparison to reference sequences. (DOCX)

## Abbreviations

DI: defective-interfering
dpi: days post infection
FBS: fetal bovine serum
H.E.: hematoxylin-eosin staining
IHC: immunohistochemistry
i.p.: intraperitoneally
MEM: minimum essential medium
MGMs: marginal zone macrophages
PFU: plaque-forming unit
rrSA51: ecombinant South Africa 1951 strain
RVF: Rift Valley fever
RVFV: Rift Valley fever phlebovirus
rZinga: recombinant Zinga strain
wt: wild-type
