# Supplementary figures and images for: Distinct virulence of Rift Valley fever phlebovirus strains from different genetic lineages in a mouse model
Source: PLoS One. 2017 Dec 21;12(12):e0189250. doi: 10.1371/journal.pone.0189250 (PMC5739399; doi:10.1371/journal.pone.0189250)

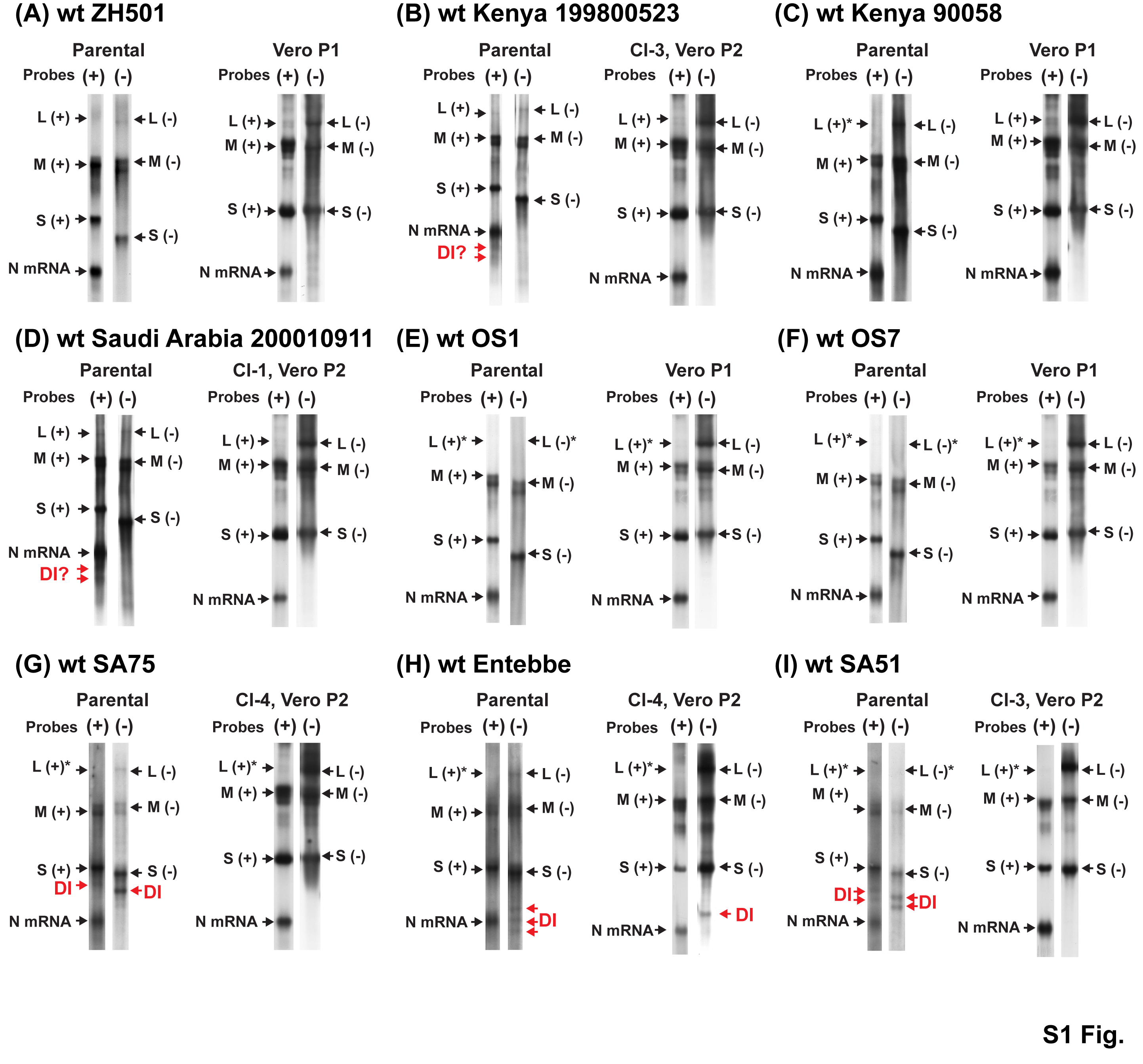

Supplement: S1 Fig — Total RNA was extracted from Vero cells infected with each stock virus (parental stock, Vero P1 of parental stock, or Vero P2 of plaque clones) at 24 hours post infection. Northern blot used RNA probes specific to antiviral-sense (+) or viral-sense (-) L, M-, or S-segment RNA. (A) wt ZH501, (B) wt Kenya 199800523, (C) wt Kenya 90058, (D) wt Saudi Arabia 200010911, (E) wt OS1, (F) wt OS7, (G) wt SA75, (H) wt Entebbe, (I) wt SA51. Defective-interfering RNA is shown in red. Probes (-) or Probes (+) represent a mixture of RNA probes detecting negative-sense or positive-sense L-, M-, and S-segments, respectively. (TIF) [file pone.0189250.s001.tif]

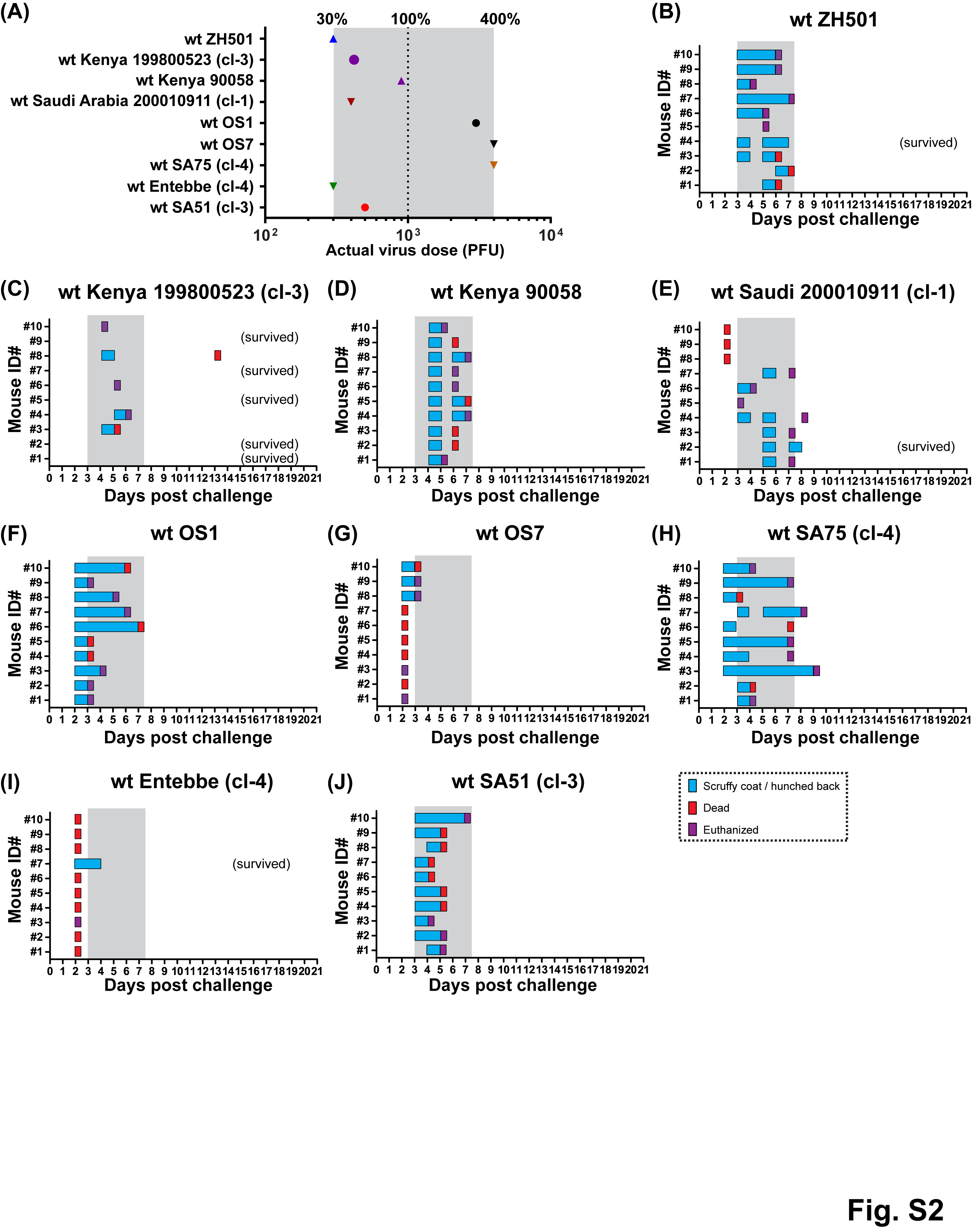

Supplement: S2 Fig — (A) Actual viral doses of wt RVFV strains; ZH501, Kenya 199800523, Kenya 90058, Saudi Arabia 200010911, OS1, OS7, SA75, Entebbe, or SA51. Percentages to the target dose (1x10^3^ PFU) are also shown. (B–K) Clinical signs of disease observed in each mouse are shown in relation to viral infection: (B) wt ZH501, (C) wt Kenya 199800523 cl-3, (D) wt Kenya 90058, (E) wt Saudi Arabia 200010911 cl-1, (F) wt OS1, (G) wt OS7, (H) wt SA75 cl-4, (I) wt Entebbe cl-4, and (J) wt SA51 cl-3. Blue = scruffy coat and/or hunched back; red = dead; purple = euthanized. (TIF) [file pone.0189250.s002.tif]

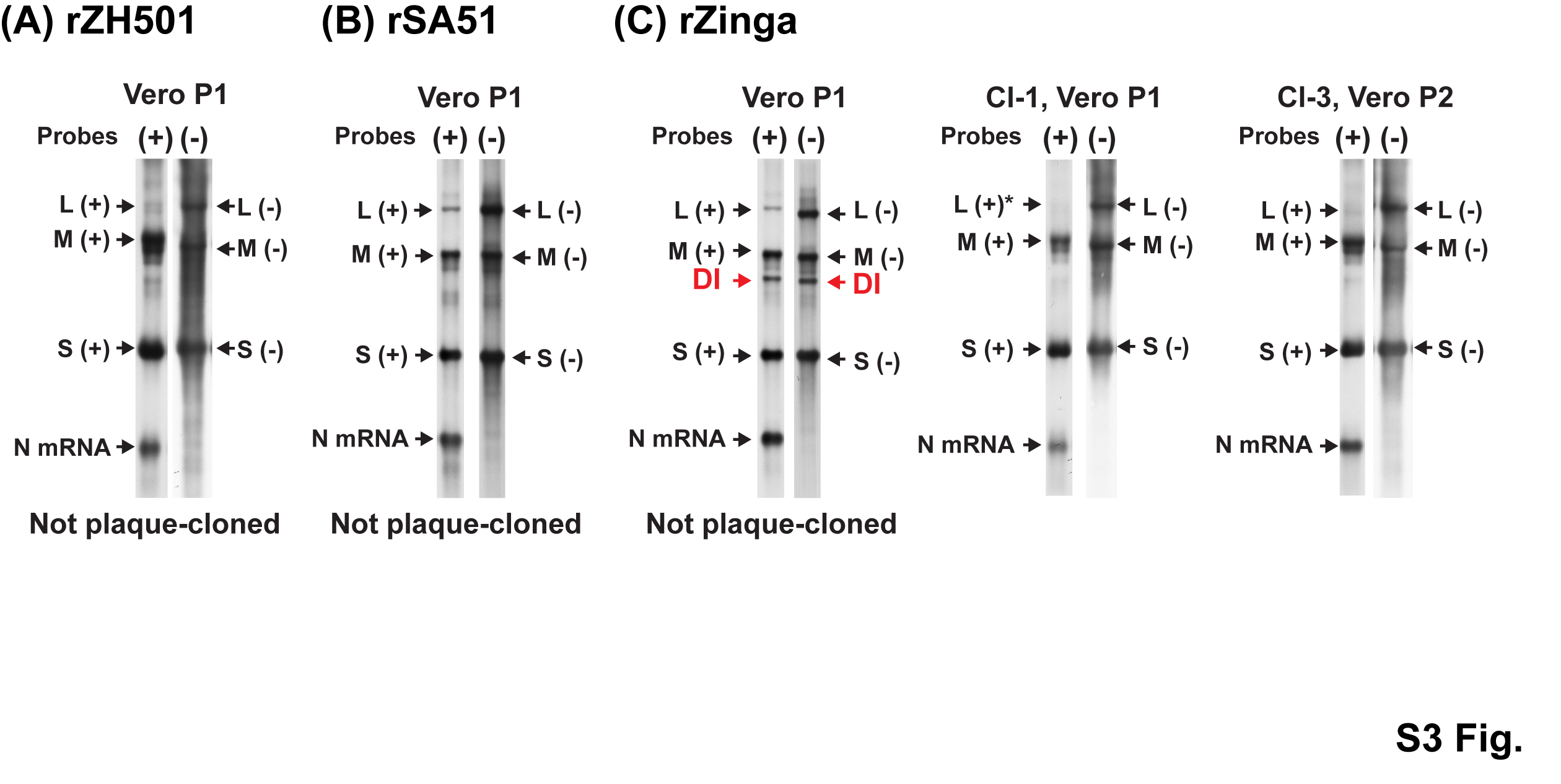

Supplement: S3 Fig — Total RNA was extracted from Vero cells infected with each stock virus (Vero P1 of rescued P0 virus or Vero P2 of plaque clones) at 24 hours post infection. Northern blot used RNA probes specific to antiviral-sense (+) or viral-sense (-) L, M-, or S-segment RNA. (A) recombinant ZH501 (rZH501), (B) recombinant SA51 (rSA51), (C) recombinant Zinga (rZinga). Defective-interfering RNA is shown in red. Probes (-) or Probes (+) represent a mixture of RNA probes detecting negative-sense or positive-sense L-, M-, and S-segments, respectively. (TIF) [file pone.0189250.s003.tif]

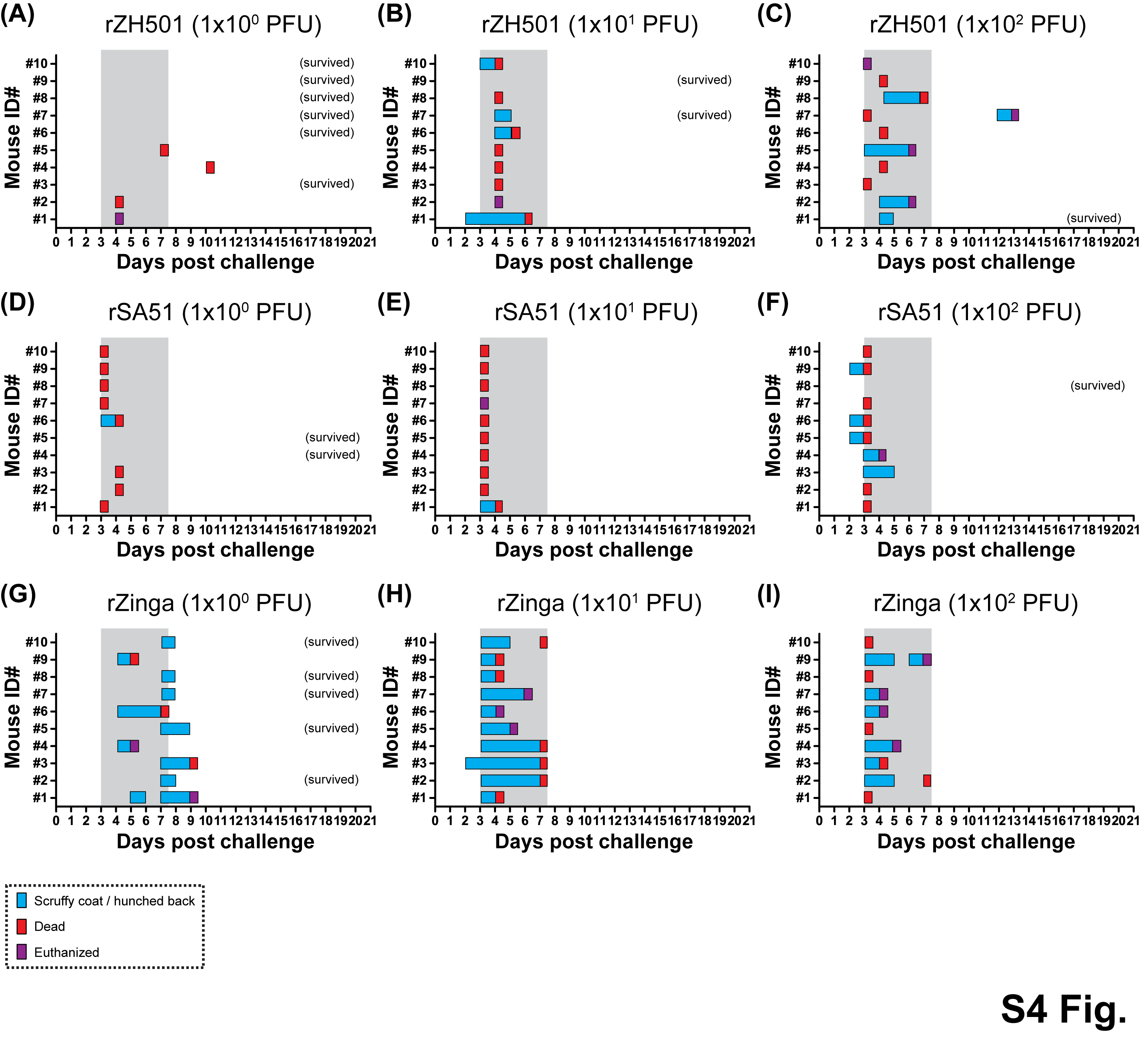

Supplement: S4 Fig — Clinical signs of disease observed in each mouse are shown in each group: (A) rZH501, 1x100 PFU, (B) rZH501, 1x101 PFU, (C) rZH501, 1x10^2^ PFU, (D) rSA51, 1x100 PFU, (E) rSA51, 1x101 PFU, (F) rSA51, 1x10^2^ PFU, (G) rZinga, 1x100 PFU, (H) rZinga, 1x101 PFU, and (I) rZinga, 1x10^2^ PFU. Blue = scruffy coat and/or hunched back; red = dead; purple = euthanized. (TIF) [file pone.0189250.s004.tif]

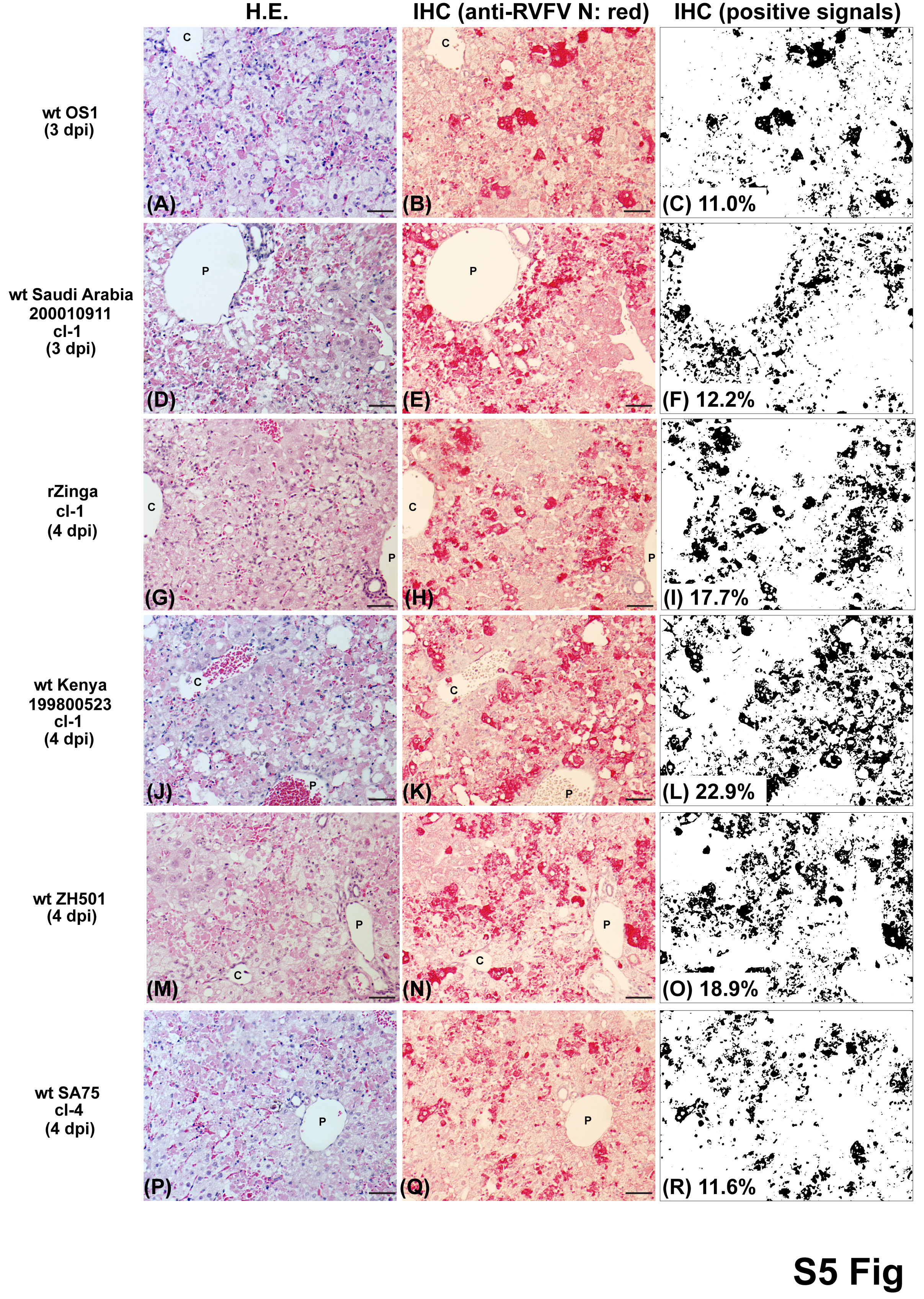

Supplement: S5 Fig — Livers of mice intraperitoneally infected with 1x10^3^ PFU of wt OS1 (A–C), wt Saudi Arabia 20010911 (D–F), rZinga (G–I), wt Kenya 199800523 (J–L), wt ZH501 (M–O), or wt SA75 (P–R) were histopathologically analyzed via hematoxylin-eosin staining (A, D, G, J, M, and P) or immunohistochemistry (IHC) using anti-RVFV N rabbit polyclonal antibody (B, E, H, K, N, and Q). The percentage of the area of positive signals was shown in each IHC image: entire the tissue area was set as 100% (C, F, I, L, O, and R). C = central vein; P = portal vein. Bars represent 50 μm. (TIF) [file pone.0189250.s005.tif]

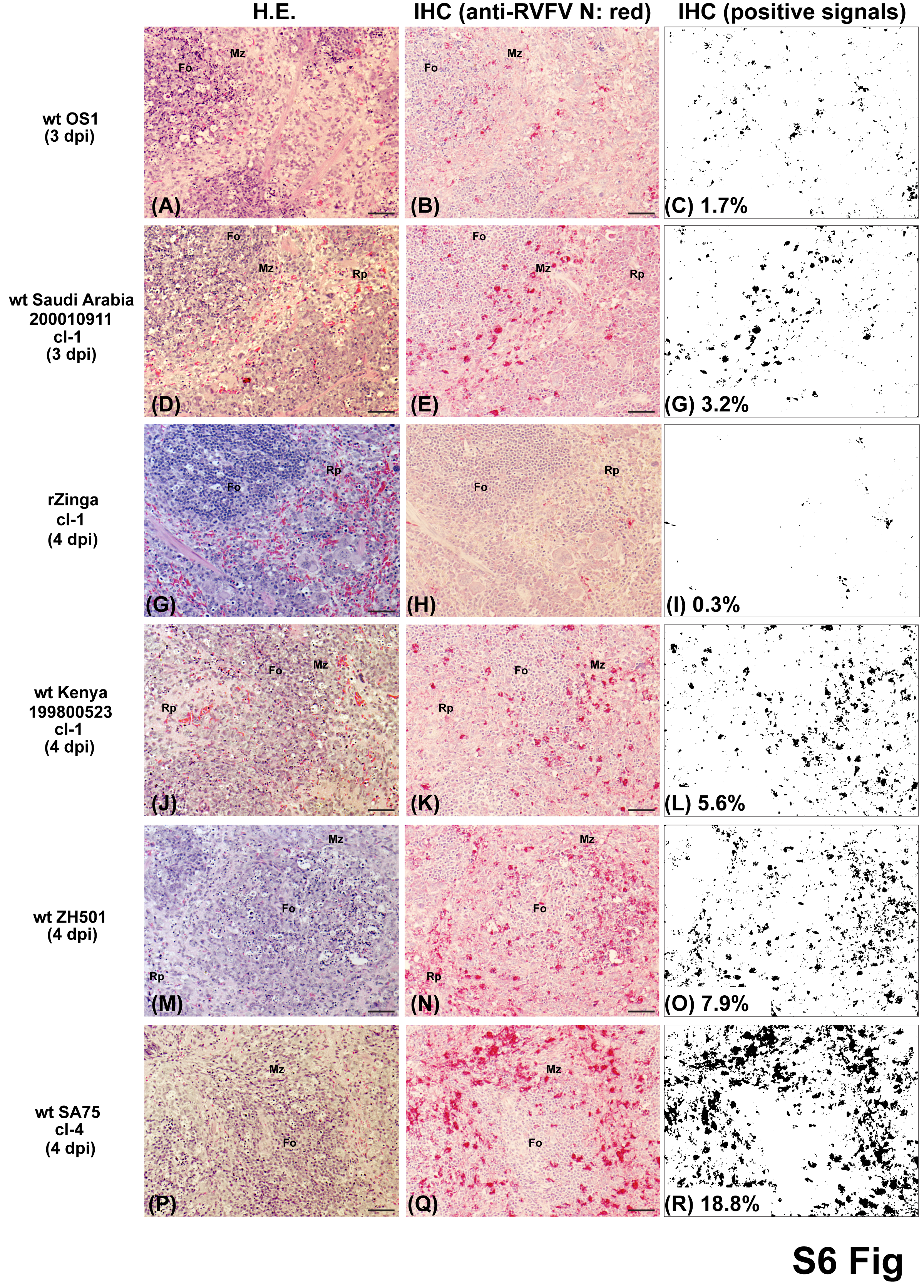

Supplement: S6 Fig — Spleens of mice intraperitoneally infected with 1x10^3^ PFU of wt OS1 (A–C), wt Saudi Arabia 20010911 (D–F), rZinga (G–I), wt Kenya 199800523 (J–L), wt ZH501 (M–O), or wt SA75 (P–R) were histopathologically analyzed via hematoxylin-eosin staining (A, D, G, J, M, and P) or immunohistochemistry (IHC) using anti-RVFV N rabbit polyclonal antibody (B, E, H, K, N, and Q). The percentage of the area of positive signals was shown in each IHC image: entire the tissue area was set as 100% (C, F, I, L, O, and R). Rp = red pulp; Fo = follicle; Mz = marginal zone. Bars represent 50 μm. (TIF) [file pone.0189250.s006.tif]

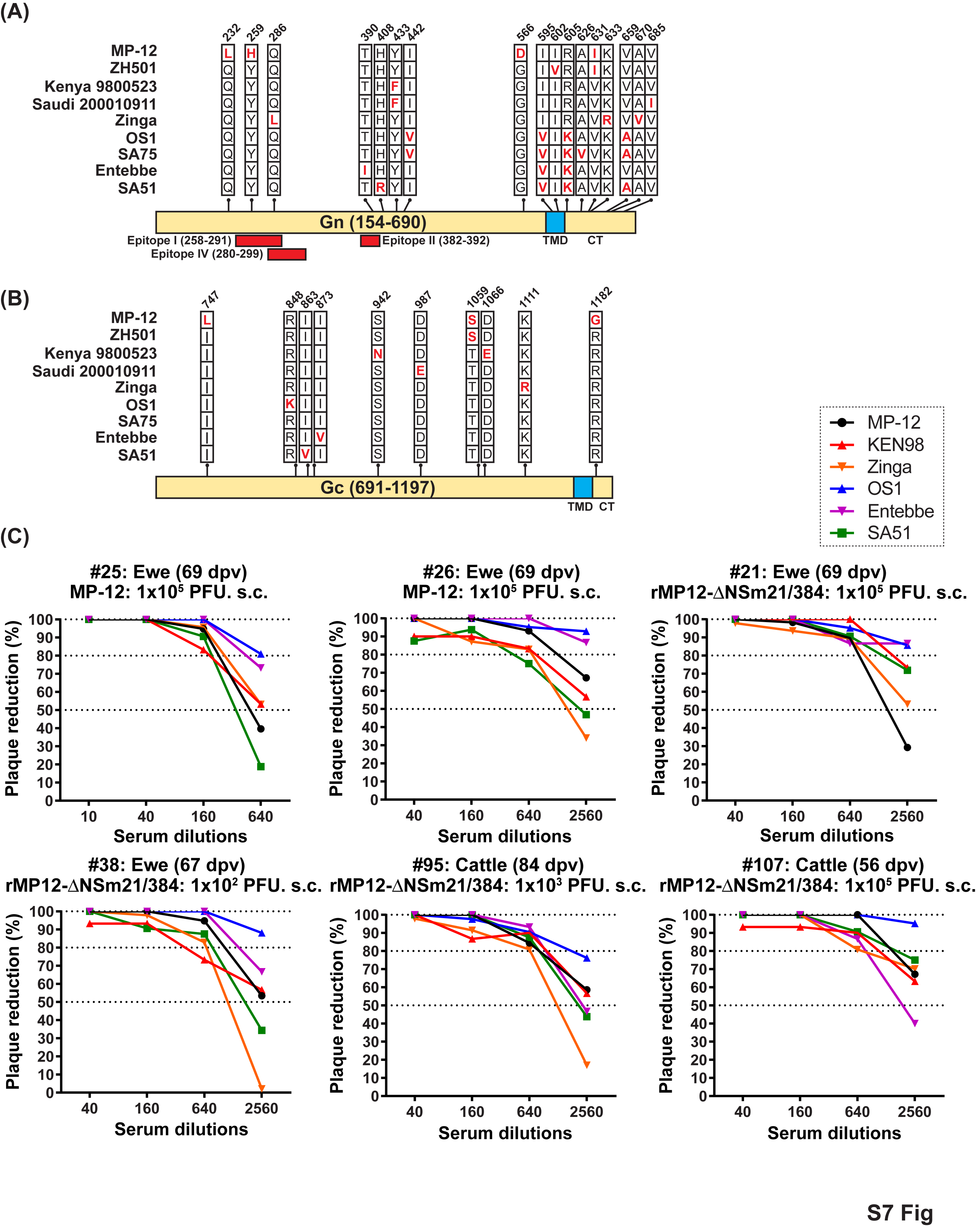

Supplement: S7 Fig — Amino acid sequences of Gn (A) or Gc (B) proteins were compared among RVFV strains MP-12 (GenBank accession DQ380208), ZH501 (DQ380200), Kenya 9800523 (DQ380196), Saudi Arabia 200010911 (DQ380197), Zinga (DQ380217), OS1 (DQ380186), SA75 (DQ380189), Entebbe (DQ380191), and SA51 (DQ380195). Amino acid positions are shown based on the precursor protein from the 1st AUG start codon in the M-segment. Locations of neutralizing epitopes I, II, and IV [33] are also shown. TMD, transmembrane domain; CT, cytoplasmic tail. (B) Serially four-fold diluted sera derived from pregnant ewes or cattle vaccinated with MP-12 or rMP12-ΔNSm21/384 [34, 35], were incubated with approximately 50 PFU of MP-12, wt Kenya 199800523, rZinga, wt OS1, wt Entebbe, or wt SA51 for the PRNT80. The reduction % of plaque numbers was calculated: the plaque number with a normal control serum was set as 100%. (TIF) [file pone.0189250.s007.tif]
