# Supplementary material for: Distinct virulence of Rift Valley fever phlebovirus strains from different genetic lineages in a mouse model
Source: PLoS One. 2017 Dec 21;12(12):e0189250. doi: 10.1371/journal.pone.0189250 (PMC5739399; doi:10.1371/journal.pone.0189250)
Supplement: S1 Table — (DOCX) [file pone.0189250.s008.docx]

**Supplementary Table 1. Sequence changes of wild-type RVFV (wt RVFV) and recombinant RVFV (rRVFV) stocks from reference sequences**

| RVFV stocks | Passage | GenBank No.  (Reference strain) | Sequence changes from parental strains |
| --- | --- | --- | --- |
| **wt RVFV** |  |  |  |
|  |  |  |  |
| **ZH501** | Vero P1 | DQ380149  DQ380200  DQ375406 | (**S**) none  (**M**) none  (**L**) none |
| **Kenya 9800523** clone 3 | Vero P2 | DQ380169  DQ380196  DQ375400 | (**S**) none  (**M**) G1195A (Gn: R392K)  (**L**) none |
| **Kenya 90058** | Vero P1 | DQ380171*  DQ380198*  DQ375402* | (**S**) none  (**M**) U3146C (silent)  (**L**) A1011G (silent), C1020U (silent) |
| **Saudi 2000-10911**  clone 1 | Vero P2 | DQ380170  DQ380197  DQ375401 | (**S**) none  (**M**) U843C (Gn: Y275H)  (**L**) C1020U (silent) |
| **OS1** | Vero P1 | DQ380180  DQ380186  DQ375398 | (**S**) none  (**M**) none  (**L**) none |
| **OS7** | Vero P1 | DQ380180**  DQ380186**  DQ375398** | (**S**) C662U (silent), C1208U (silent)  (**M**) G303A (NSm: D95N), G755A (silent), U2135C (silent), G3707A (5’UTR), G3794A (5’UTR)  (**L**) C720U (silent) |
| **SA75**  clone 4 | Vero P2 | DQ380175  DQ380189 DQ375428 | (**S**) A514G (N: E159G)  (**M**) A864G (Gn: N282D)  (**L**) C1020U (silent), C1025U (A336V) |
| **Entebbe**  clone 4 | Vero P2 | DQ380156  DQ380191  DQ375429 | (**S**) A764G (silent)  (**M**) 3662_3663insA (insertion: 5’UTR)  (**L**) A4435G (I1473V), C5446U (P1810S) |
| **SA51**  clone 3 | Vero P2 | DQ380158  DQ380195  DQ375433 | (**S**) none  (**M**) A774G (Gn: N252D)  (**L**) A851G (N284S), A1426C (N476H) |
| **rRVFV** |  |  |  |
|  |  |  |  |
| **rSA51** | Vero P1 | DQ380158  DQ380195  DQ375433 | (**S**) none  (**M**) none  (**L**) C1020U (silent) |
| **rZinga**  clone 1 | Vero P2 | DQ380167  DQ380217  DQ375419 | (**S**) A685G (N: E216G)  (**M**) none  (**L**) none |
| **rZinga**  clone 3 | Vero P2 | DQ380167  DQ380217  DQ375419 | (**S**) A1004C (NSs: L218R)  (**M**) none  (**L**) A1100G (E361G) |
| * Kenya 1983 21445 strain sequences were used for the alignment with Kenya 90058. | | | |
| ** OS1 strain sequences were used for the alignment with OS7.  The L-, M-, and S-segment sequences of RVFV Kenya 90058 and OS7 strains were deposited in the GenBank (accession numbers: MG273455 – MG273460). | | | |
